# Maternal plasma levels of oxytocin during physiological childbirth – a systematic review with implications for uterine contractions and central actions of oxytocin

**DOI:** 10.1186/s12884-019-2365-9

**Published:** 2019-08-09

**Authors:** Kerstin Uvnäs-Moberg, Anette Ekström-Bergström, Marie Berg, Sarah Buckley, Zada Pajalic, Eleni Hadjigeorgiou, Alicja Kotłowska, Luise Lengler, Bogumila Kielbratowska, Fatima Leon-Larios, Claudia Meier Magistretti, Soo Downe, Bengt Lindström, Anna Dencker

**Affiliations:** 1 University of Agriculture (SLU), Uppsala, Sweden; 2 School of Health and Education, University of Skövde, Skövde, Sweden; 3 Department of Health Sciences, University West, Trollhättan, Sweden; 4 Institute of Health and Care Sciences, University of Gothenburg, Gothenburg, Sweden; 5 Centre for Person-Centred Care, University of Gothenburg, Gothenburg, Sweden; 6 School of Public Health, The University of Queensland, Brisbane, Australia; 7 Faculty of Health Sciences, Oslo and Akershus University College of Applied Sciences, Oslo, Norway; 8 Faculty of Health Sciences, Cyprus, University of Technology, Limassol, Cyprus; 9 Faculty of Health Sciences with Subfaculty of Nursing and Institute of Maritime and Tropical Medicine, Medical University of Gdańsk, Gdańsk, Poland; 10 Midwifery Research and Education Unit, Hannover Medical School, Hannover, Germany; 11 Faculty of Medical Sciences, Medical University of Gdańsk, Gdańsk, Poland; 12 Faculty of Nursing, Physiotherapy and Podiatry, University of Seville, Seville, Spain; 13 Department of Social Work Center for Health Promotion and Social Participation, Lucerne University of Applied Sciences and Arts, Luzern, Switzerland; 14 Research in Childbirth and Health (ReaCH) group, University of Central Lancashire, Preston, UK; 15 Norwegian University of Science and Technology, Trondheim, Norway

**Keywords:** Oxytocin, Plasma levels, Pregnancy, Physiological labour, Birth, Uterine contractions, Central effects, Neurobiology, Infusion of synthetic oxytocin

## Abstract

**Background:**

Oxytocin is a key hormone in childbirth, and synthetic oxytocin is widely administered to induce or speed labour. Due to lack of synthetized knowledge, we conducted a systematic review of maternal plasma levels of oxytocin during physiological childbirth, and in response to infusions of synthetic oxytocin, if reported in the included studies.

**Methods:**

An a priori protocol was designed and a systematic search was conducted in PubMed, CINAHL, and PsycINFO in October 2015. Search hits were screened on title and abstract after duplicates were removed (*n* = 4039), 69 articles were examined in full-text and 20 papers met inclusion criteria. As the articles differed in design and methodology used for analysis of oxytocin levels, a narrative synthesis was created and the material was categorised according to effects.

**Results:**

Basal levels of oxytocin increased 3–4-fold during pregnancy. Pulses of oxytocin occurred with increasing frequency, duration, and amplitude, from late pregnancy through labour, reaching a maximum of 3 pulses/10 min towards the end of labour. There was a maximal 3- to 4-fold rise in oxytocin at birth. Oxytocin pulses also occurred in the third stage of labour associated with placental expulsion. Oxytocin peaks during labour did not correlate in time with individual uterine contractions, suggesting additional mechanisms in the control of contractions. Oxytocin levels were also raised in the cerebrospinal fluid during labour, indicating that oxytocin is released into the brain, as well as into the circulation. Oxytocin released into the brain induces beneficial adaptive effects during birth and postpartum.

Oxytocin levels following infusion of synthetic oxytocin up to 10 mU/min were similar to oxytocin levels in physiological labour. Oxytocin levels doubled in response to doubling of the rate of infusion of synthetic oxytocin.

**Conclusions:**

Plasma oxytocin levels increase gradually during pregnancy, and during the first and second stages of labour, with increasing size and frequency of pulses of oxytocin. A large pulse of oxytocin occurs with birth. Oxytocin in the circulation stimulates uterine contractions and oxytocin released within the brain influences maternal physiology and behaviour during birth. Oxytocin given as an infusion does not cross into the mother’s brain because of the blood brain barrier and does not influence brain function in the same way as oxytocin during normal labour does.

**Electronic supplementary material:**

The online version of this article (10.1186/s12884-019-2365-9) contains supplementary material, which is available to authorized users.

## Plain English Summary

Oxytocin is an important hormone in labour and birth, when it helps the labouring woman’s uterus to contract and birth her baby. We searched for all the studies that measured blood levels of oxytocin in women during normal (physiological) labour and birth.

We found that blood oxytocin levels gradually rise in pregnancy and become even higher during labour, when pulses of oxytocin become progressively bigger and more frequent. Oxytocin contracts the uterus and promotes the progress of labour. A large oxytocin pulse occurs with the birth, and pulses continue afterwards, which help the new mother to birth the placenta, prevent bleeding, and warm her chest for skin-to-skin contact with her baby.

During labour, oxytocin is released into both the blood and brain, with high oxytocin levels in the brain fluid (cerebrospinal fluid, CSF). Oxytocin has many positive effects in the mother’s brain during labour, and prepares her for motherhood. Oxytocin reduces anxiety, stress and pain in labour and switches on brain pleasure and reward centres, making the new mother relaxed, and happy as she meets her baby for the first time.

Several studies measured oxytocin levels when infusion of synthetic oxytocin was used for induction or augmentation of labour. These studies found that low doses of synthetic oxytocin produced similar oxytocin levels as physiological labour whereas high doses gave abnormally high oxytocin levels, in proportion to dosage. Labour induced by synthetic oxytocin differs in some way from normal, physiological labour. It may, especially at high dosages cause more, longer and more painful contractions, when compared to normal labour. Oxytocin given as an infusion does not cross into the mother’s brain because of the blood brain barrier and does not influence brain function in the same way as normal labour does.

## Background

The peptide hormone oxytocin was discovered in 1906 by Sir Henry Dale, who found that a pituitary extract stimulated uterine contractility in cats [1]. The chemical structure of oxytocin was elucidated in the 1950s by Vincent du Vigneaud and oxytocin was synthesized soon after [2].

It is well known that oxytocin plays a pivotal role during human labour and birth.

Oxytocin is produced in neurons that originate in the paraventricular (PVN) and supraoptic (SON) nuclei of the hypothalamus, and is transported to the posterior pituitary. During labour, oxytocin is released in pulses from the pituitary into the circulation to induce uterine contractions [3].

Research interest in oxytocin has increased dramatically in recent years, as oxytocin has been found to have many positive physiological and psychological effects [4]. This is due to the fact that oxytocin is released within the brain. Oxytocinergic nerves originating in the PVN project to many important regulatory areas, where oxytocin is released to act as a neuromodulator, with widespread central effects [5]. For example, oxytocin enhances mood and wellbeing; promotes friendly social interactions; reduces anxiety and pain; and lowers physiological and psychological stress, among other benefits [6]. In addition, it reduces sympathetic nervous system activity (“fight or flight”), and increases parasympathetic nervous system activity (“relaxation and growth” and “calm and connection”) [4]. Given this information on oxytocin, and its established role in maternal behaviours in all mammals [7], it is important to consider the possible effects of oxytocin released in the brain during childbirth.

In addition, infusions of synthetic oxytocin are widely used in maternity care to induce or augment labour, and are also recommended to prevent or treat postpartum bleeding [8]. Because of the widespread and increased use of synthetic oxytocin, it is critical to understand the possible impacts on endogenous oxytocin, and on oxytocin-mediated effects in mothers and babies [9, 10].

As part of a collaborative European project initiated within the European COST program IS1405, we aim at performing several systematic reviews summarizing existing data on plasma levels and effects of oxytocin in mothers, during and after physiological childbirth, including longer-term effects on breastfeeding, attachment, and mental health, and in women with and without medical interventions. Recent information on the beneficial central effects of oxytocin must be incorporated into the present knowledge about the role of oxytocin during physiological labour.

Previous studies that measured oxytocin levels during birth were mainly published in the 1970s to 90s. Most of these older studies are of high quality and provide an accurate assessment of oxytocin levels, and would be difficult to perform today because of technical, practical and ethical reasons. It is of importance to bring back this forgotten but highly relevant knowledge to healthcare professionals of today involved in labour and birth. In addition, there are many misunderstandings regarding the effects of endogenous oxytocin and exogenous synthetic oxytocin during childbirth that could be resolved by access to this knowledge, with benefits for clinical care.

The specific aim of this systematic review is to summarize existing data on plasma levels of oxytocin during normal physiological birth. In addition, data from the reviewed studies on plasma levels of oxytocin after infusions of synthetic oxytocin will be included. This will allow comparison between oxytocin levels observed during physiological birth and in birth induced or augmented with synthetic oxytocin. We will also discuss the possible central effects of oxytocin released in the brain during physiological birth.

## Methods

A systematic review was carried out according to the PRISMA statement [11]. An a priori protocol was designed, outlining the aim and procedure for the review. *Inclusion criteria* were studies presenting blood/plasma oxytocin levels in women having had a normal pregnancy without medical complications, a physiological birth, and who had given birth to a healthy baby at term. Included were peer-reviewed original research studies comprising at least one measure of plasma oxytocin levels in women during a physiological labour, and which were written in either English, French or German as these were the languages available to the review team.

### Search strategy

A comprehensive and systematic search was done in the following databases: PubMed, CINAHL and PsycINFO in October 2015, to identify studies or existing systematic reviews. We also hand-searched the reference lists of all eligible publications for references to other possibly relevant studies. The search string is available in the Additional file 1.

**Fig. 1:**
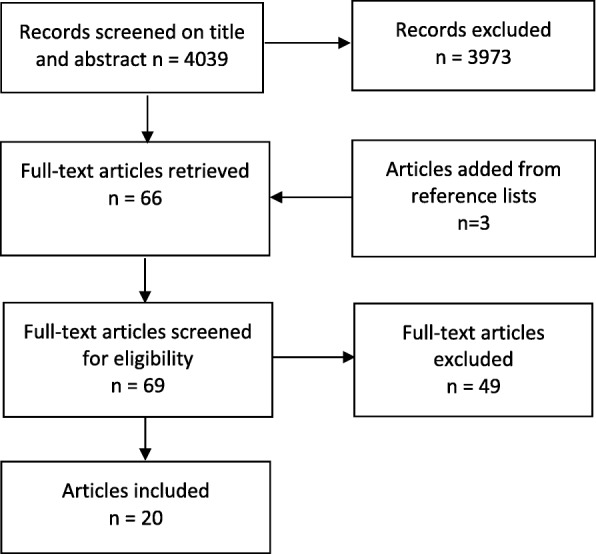
Flow diagram of study selection

A total of 4039 publications were found after removal of duplicates. Assessment of titles and abstracts were divided in six groups of authors (KUM & AEB; AK& BK; MB & LL; EH & CMM; FLL & ZP; AD & SD) and assessed independently by the authors in pairs. The assessment was based on the inclusion criteria, i.e. women without medical complications during pregnancy or labour. In case of disagreement a third reviewer (KUM) was involved. This process ended in exclusion of 3973 papers. The full text of the remaining papers (*n* = 66) was read by a team of two researchers (KUM and AEB). Three further studies were added from reference lists giving 69 papers which were screened for eligibility. Of these studies 49 were excluded because they did not fulfil the inclusion criteria. This process led to a final number of 20 publications to be included for data extraction. For overview of study selection see the flow diagram in Fig. 1.

### Data extraction and analysis

A combined analysis of all data was not possible because the studies differed substantially as to the type of biochemical analysis used to determine oxytocin levels as to when during labour, as well as to the timing and the frequency of blood sampling (single samples, repeated samples with different time intervals including months, weeks, hours, minutes or seconds). We therefore created a narrative synthesis for each article. From these narrative results, oxytocin levels during labour were extracted and then summarized to allow characterization of oxytocin levels during the different time periods of birth, and also comparison between groups.

Some of the included studies also contained information about plasma levels of oxytocin during basal conditions (non-pregnant women) and during pregnancy. These data were included to allow comparison between oxytocin levels in these situations and plasma levels of oxytocin during birth. Information about oxytocin levels in cerebrospinal fluid (CSF) during pregnancy and birth was also included. Data on oxytocin levels following bolus injections or infusions of synthetic oxytocin described in the included studies are also presented. Finally, data on the association between uterine contractions and plasma levels of oxytocin were included. In some studies, oxytocin levels were measured both before and after medical interventions. In all except one of the included studies, radioimmunoassay (RIA) was used to determine oxytocin levels [12].

The levels of oxytocin in the 20 selected papers were presented in different measures/units, e.g. μU/mL, pg/mL or pmol/L (pM). In order to facilitate interpretation of the results, Table 1 shows conversion between international units (IU), weight units (g) and molar units (M). All samples were from peripheral veins, except one study that simultaneously also collected blood from the jugular vein.

**Table 1:** Conversion of oxytocin levels between units

Conversion of different size units:	
1 Unit (IU) = 1000 milliunits (mU) = 1000,000 microunits (μU)	
1 g (g) = 1000 mg (mg) = 1000,000 micrograms (μg) = 1000,000,000 nanograms (ng) = 1000,000,000,000 picograms (pg)	
1 Mol/L = 1 Molar (M) = 1000 milliMolar (mM) = 1000,000 microMolar (μM) = 1000,000,000 nanoMolar (nM) = 1000,000,000,000 picoMolar (pM)	
Conversion of units (U) to weight units:	
1 IU = 1.67 μg, 1 μg = 0.60 U	
1 mU = 1.67 ng, 1 ng = 0.60 mU	
1 μU = 1.67 pg, 1 pg = 0.60 μU	
Conversion of weight/volume of oxytocin to Molarity (molecular weight of oxytocin equal to 1007 g/mol):	
1 g/mL = 1 mol/L = 1 M	
1 mg/mL = 1 mmol/L = 1 mM	
1 μg /mL = 1 μmol/L = 1 μM	
1 ng/mL = 1 nmol/L = 1 nM	
1 pg/mL = 1 pmol/L = 1pM	

## Results

As described, 20 articles that measured oxytocin levels during labour and birth met the criteria for inclusion [13–32]. The studies were published between 1965 and 2001. In addition to measurement of oxytocin levels in labour, oxytocin levels were also measured during pregnancy in 10 of the 20 included studies. Some studies also included measures of oxytocin levels in non-pregnant women. Characteristics of the included articles are reported in Table 2.

**Table 2:** Characteristics of included studies

First author, year, ref. no.	Data collection	Methodology	Comment
Coch 1965 [13]	Blood samples from the jugular and a peripheral vein at various stages of labour and postpartum, in 18 women.	Bioassay^a^	Bioassay based on milk ejection in rabbits calculating “oxytocin equivalent activity.” Very high levels compared to modern assays using RIA.
Kumaresan 1974 [14]	Single blood samples between weeks 4 and 40 during pregnancy in 280 women. 79 random samples during active labour in 5 women. Serial samples were obtained before and after oxytocin infusion of 100 mU per min to women with mid-pregnancy terminations.	RIA^b^	OT determinations performed with RIA without prior extraction, explaining why high levels. OT levels were already very high at term, but did not rise further during labour, maybe due to an insensitivity of this assay at high levels of OT.
Kumaresan 1975 [15]	Single maternal blood samples were collected from 29 women 10 min before birth, just after birth and daily for 4 days postpartum.	RIA^b^	OT determinations performed with RIA without prior extraction, giving higher levels. Lack of a significant peak at birth suggests possible insensitivity of the assay at high levels.
Gibbens 1976 [16]	8 serial blood samples from 97 women during spontaneous labour (1st stage 33, 2nd stage 14, 3rd 10). Single samples from a further 30 women during the 3rd stage of labour. The serial samples were collected over a period of 4 to 8 min.	RIA^c^	Pulsatile release of OT was detected. Very low basal levels of OT may be due to insensitivity of the RIA used, or to a loss of OT during the extraction procedure.
Vasicka 1978 [17]	Blood samples were collected in a longitudinal study (during pregnancy, onset of labour and birth) in 15 women. Samples at one- to two-week intervals during pregnancy and one minute to one hour intervals throughout labour and birth.	RIA^b^	OT determinations performed with RIA without prior extraction, explaining high levels*.*
Dawood 1979 [18]	362 blood samples were collected from normal pregnant women. Serial blood samples of blood were obtained from 10 pregnant women through gestation until labour onset. Serial samples taken at 1 min intervals over a 10 min period in 7 pregnancies and 3 in 1st stage of labour.	RIA^c^	OT levels recorded within the range normally observed with established RIA after prior extraction of the samples.
Leake 1981 [19]	Plasma oxytocin levels in 102 non-pregnant women, 20 women receiving oral contraceptive medications and 59 pregnant women from 15 to 42 w of pregnancy. Repeated samples were collected in 38 healthy women during spontaneous labour.	RIA^c^	Very low OT levels. RIA did not pick up differences between pregnant and non-pregnant women; the rise of oxytocin occurring during pregnancy; or the rise of OT observed during labour, suggesting that the RIA used was very insensitive. Significant rise of OT level observed was in connection with birth only.
Otsuki 1983 [20]	Individual samples from 38 normal pregnant women between the 15th and the 41 st w of pregnancy. Serial samples from 2 women in the mid trimester, from 4 women at term without labour contractions and from 6 women in the 1st stage of normal spontaneous labour, at 10 s intervals over a period of 2–3 min.	RIA^c^	Relevant OT levels within the range normally observed with established RIAs.
Goodfellow 1983 [21]	20 primigravidae with normal labour, after 37 to 41 weeks of pregnancy. Epidural analgesia was chosen by half of the women. Blood samples at the beginning and end of 2nd phase of labour.	RIA^c^	Relevant OT levels within the range normally observed with established RIA.
Husslein 1983 [22]	20 women with spontaneous labour. Blood samples were collected just before the 2nd phase of labour, 5 min postpartum, 30 min after expulsion of placenta and 2 h after birth. 7 women received oxytocin drip in a low dosage at the end of the first phase of labour. Directly after birth 10 women received oxytocin drip in a high dosage (100-150mIU/min).	RIA^c^	Relevant OT levels giving values within the range normally observed with established RIA.
Fuchs 1983 [23]	17 women in early spontaneous labour and 15 women at term who were given oxytocin induction. Five blood samples. In one group at admission to the labour ward and subsequent samples were taken at 1–3 h intervals. In another group a blood sample just before infusion of oxytocin was started and thereafter just before the infusion rate was increased. Infusion was begun at the rate of 1–2 mU/min and increased stepwise every 15 min until contractions occurred with about 3 min intervals. Thereafter the infusion rate was kept constant. 10 of these 17 women contributed a blood sample 1–2 w before labour. The control group consisted of 4 pregnant women at term but not in labour. Four serial samples were taken at 2 h intervals from each woman.	RIA^c^	Relevant OT levels within the range of values normally observed with established RIA.
Amico 1984 [24]	Eleven women with “hypocontractile labour” received oxytocin infusion in a dose of 1 mU/min and it was increased by one 1 mU every 40 min until adequate contractions were observed. Blood samples were drawn before start of infusion and at every 20 min, during infusion and for 60 min after the end of infusion.	RIA^c^	Very low basal levels of OT maybe due to insensitivity of the RIA used or to loss of OT during the extraction procedure.
Takeda 1985 [25]	42 participants were included in the study, 4 healthy males, 15 non-pregnant women, and 23 pregnant women (11 before and 12 in labour). Blood and cerebrospinal fluid (CSF) samples were collected simultaneously from all the participants.	RIA^c^	Relevant OT levels within the range of values normally observed with established RIA.
Takagi 1985 [26]	36 women were included in the study (7 non pregnant women, 11 pregnant women having an emergency CS and 18 pregnant women having an elective CS. 1 blood and cerebrospinal fluid (CSF) samples were collected simultaneously from all the women	RIA^c^	Relevant oxytocin levels within the range of values normally observed with established RIA.
De Geest 1985 [27]	10 pregnant women and 15 women during normal labour, 5 of which received EDA. Four blood samples during pregnancy and during the 1st and 2nd stage of labour. Blood samples were also collected from umbilical arterial and venous blood vessels after birth	RIA^b^	OT determinations performed with RIA without prior extraction, which explains high OT levels*.* A rise of OT level observed during pregnancy, but not during labour, suggesting that the RIA used was insensitive at higher levels than those observed during pregnancy.
Kuwabara 1987 [28]	Repeated blood samples in 6 normal pregnant women and blood samples were taken in 7 normal pregnant women every 2 days for at least 14 days before the onset of labour. Simultaneous blood samples were collected from maternal venous blood, umbilical arterial and venous vessels in 10 normal deliveries, 15 elective caesarean sections, and 5 emergency caesarean sections. Amniotic fluid samples were collected during pregnancy and in elective caesarean sections. Blood samples were also collected from 10 non-pregnant women.	RIA^c^	Relevant OT levels within the range of values normally observed with established RIA.
Thornton 1988 [29]	25 women having spontaneous labour, of these 10 women received synthetic OT i.m. when the anterior shoulder was delivered. Blood samples were collected every 30th second (for 15 min) after crowning of the head.	RIA^c^	Low, but relevant OT levels within the range of values observed with established RIA.
Oosterbaan 1989 [30]	Maternal venous blood was collected at the time of amniocentesis (*n* = 17), elective CS (*n* = 18) and/or immediately after normal birth of the baby (*n* = 44). Mixed cord blood or blood from the umbilical artery or vein was collected at the time of elective CS or after spontaneous birth of the baby.	RIA^c^	Relevant OT levels within the range of values normally observed with established RIA.
Fuchs 1991 [31]	50 pregnant women (38 to 42 weeks of gestation) Samples were collected with 1 min intervals for 30 min from the following groups: 1) Women at term who were scheduled for elective CS, with a closed cervix and not in labour (*n* = 11). 2) Women in the 1st stage of spontaneous labour with < 6 cm cervical dilation (*n* = 13) 3) Women in the 2nd stage of spontaneous labour with full dilatation of the cervix (*n* = 8). Five of these women delivered during the 30 min long sampling period and therefore samples were collected also after birth until delivery of the placenta. 4) 18 women who were not in labour were give bolus injections of OT iv, 2, 4, 8 or 16 mU. 8 blood samples before and 30 s, 1, 2, 3, 4, 5 and 10 min after injection (n = 18).	RIA^d^	Very low basal levels of OT perhaps due to insensitivity of the RIA used or a loss of OT during the extraction procedure. Frequent sampling of blood, together with low basal levels of OT, allowed recording and quantitative analysis of individual OT pulses.
Stocche 2001 [32]	30 women included in a randomized open label. Each women was in spontaneous labour at > 5 cm cervix dilatation. Patients received either intrathecal sufentanil 10 microgram or epidural plain bupivacaine 0.25% Serial blood samples were collected before analgesia and 15, 30, 60 and 90 min after the induction of the analgesia.	RIA^c^	Low, but relevant OT levels within the range of values observed with established RIA.

*CS* caesarean section, min = minute/s, hrs = hours, w = weeks, EDA = epidural analgesia, OT = oxytocin

^a^Oxytocin like activity was measured by bioassay

^b^Radioimmunoassay performed on unextracted plasma

^c^Radioimmunoassay performed on extracted plasma

^d^Radioimmunoassay performed on extracted plasma. Note that the antibody does not, measure the type of oxytocin released during pregnancy

All oxytocin levels converted to pg/mL are summarized in Table 3. The results obtained after extraction of data from the individual articles were summarized into categories, which are presented as follows; “Summary of oxytocin levels during labour and birth” (Table 4); “Summary of oxytocin levels during pregnancy and in non-pregnant women” (Table 5) and “Summary of the occurrence of uterine contractions in relation to plasma levels of oxytocin during labour without and with synthetic oxytocin” (Table 6).

**Table 3:** Oxytocin levels in blood samples collected during physiological birth in the 20 selected papers. Values from pregnant and non-pregnant women are included. All values are presented in pg/mL, with SD or range in parentheses

First author, year	Labour and birth (pg/mL)	Time for blood sample	Pregnancy (pg/mL)	Gestational week (w)	Non-pregnant (pg/mL)
Coch 1965 [13]^a^	167	Active labour			
Kumaresan 1974 [14]^a^	302 (58.3–725.0)	Active labour	110	4 w	< 1.7
			275	40 w	
Kumaresan 1975 [15]^a^	150.0 (18.3)	10 min before birth			
	136.7 (20.0)	Immediately after birth			
Gibbens 1976 [16]^a^	4.2–20.8	1st – 3rd stage			
Vasicka 1978 [17]^a^	73.3–273.3	Active labour	211.5 (35.2)	24 w	
			255.0 (44.3)	40 w	
Dawood 1979 [18]	0–86	1st stage of labour	17.9 (2.1)	1–20 w	
			28.9 (4.7)	21–30 w	
			32.9 (2.9)	31–32 w	
			26.4 (4.8)	38 w	
			74.2 (14.2)	39 w	
Leake 1981 [19]^a^	2.2 (0.3	Latent phase	2.2 (0.2)	15–42 w	2.3 (0.3)
	2.7 (0.3)	Active labour			
	1.8 (0.2)	Visible head			
	7.0 (1.8)	Delivery of head			
Otsuki 1983 [20]^a^	21.0 (63.3–146.7)	Active labour	8.3	20 w	
			20.8 (12.8)	36–41 w	
Goodfellow 1983 [21]	7.2–46.0;	Full dilatation			
	8.8–85.0^c^	Crowning of head			
Husslein 1983 [22]	50.7 (10.7) 29.1 (9.7)	Full dilatation 30 min postpartum			
Fuchs 1983 [23]	45 (3.9) 21.1 (6.4) 49.1 (10.9) 58.8 (9.9) 110 (22.7)	1st stage 1–3 mU/min SOT inf 4–6 mU/min SOT inf 7–9 mU/min SOT inf 10–15 mU/min SOT inf	15.4 (8.7) 17.4 (4.8) 19.9 (3.1)	1–2 weeks before labour Before induction At term	
Amico 1984 [24]^a^	1.7 (0.5)	Arrest in active labour			
Takeda 1985 [25]^a^	32.8 (5.5)	Active labour	26.8 (4.8)	7–41 w	8.0 (0.8)
Takagi 1985 [26]^a^	75.3 (32.7)	Before emergency CS			11.7 (8.8)
De Geest 1985 [27]^a^	276.7	< 3 cm cx dilatation	78.3	8–12 w	
	298.3	5–7 cm	128.3	20–24 w	
	281.7	10 cm	208.3	30–34 w	
	276.7	2nd stage	226.7	38–42 w	
Kuwabara 1987 [28]^a^	51.0 (8.3)	Immediately after birth	27–28	6–41 w	6.8 (0.8)
			33.3	At term	
Thornton 1988 [29]^b^	3.2 (2)	Crowning of head			
	6.4 (2)	After delivery of shoulder			
	11.6	Mean peak after birth			
Oosterbaan 1989 [30]	65 (9) 12 (9)	Immediately after birth During prelabour CS			
Fuchs 1991 [31]^a^	1.8 (0.2)	1st stage < 6 cm	1.5 (0.5)	At term before elective CS	
	2.3 (0.6)	2nd and 3rd stage			
Stoche 2001 [32]	7.4 (2.1)	1st stage			
	6.6 (3.1)	Before analgesia			

*pg/mL* picograms per millilitre, *mU/min* milliunits per minute, *SOT inf* synthetic oxytocin infusion, *CS* caesarean section, *μU/mL* microunits per millilitre, *pmol/L* picomoles per litre

^a^Oxytocin levels have been converted from μU/mL to pg/mL

^b^Oxytocin levels have been converted from pmol/L (pM) to pg/mL

^c^Corrected from Table 1 in Goodfellow 1983

**Table 4:** Summary of oxytocin levels during labour and birth (converted to pg/mL)

Oxytocin levels varied significantly between the studies. Very low values around 1.7–3.4 pg/mL were reported by: Leake 1981 [19], Amico 1984 [24], and Fuchs 1991 [31]. Very high levels, around 167–340 pg/mL, were reported by Coch 1965 [13], Kumaresan 1974 [14] and 1975 [15], Vasicka 1978 [17], and De Geest 1985 [27]. These high levels were obtained using bioassay or RIA without previous extraction of the plasma samples, which likely explains the higher-than-expected results. The majority of the articles report the levels as expected, between 17 and 85 pg/mL; Gibbens 1976 [16], Dawood 1979 [18], Otsuki 1983 [20], Goodfellow 1983 [21], Husslein 1983 [22], Fuchs 1983 [23], Takeda 1985 [25], Takagi 1985 [26], Kuwabara 1987 [28], Thornton 1988 [29], Oosterbaan 1989 [30], and Stoche 2001 [32]. *Difference in oxytocin levels between pregnancy and labor* A rise of oxytocin levels between pregnancy and labor was observed by Dawood 1979 [18], Husslein 1983 [22], Fuchs 1983 [23], and Fuchs 1991 [31]. No rise was observed by Kumaresan 1974 [14] and 1975 [15], and De Geest 1985 [27], which was most likely due to a lack of sensitivity of the assay used. *Difference in oxytocin levels over time during labor, or between the 1st and the 2nd stage of labor* A rise of oxytocin levels during labor was observed by Coch 1965 [13] and Gibbens 1976 [16]. In addition, Dawood 1979 [18], Otsuki 1983 [20], and Fuchs 1991 [31] found an increasing incidence, frequency, and/or amplitude of oxytocin pulses, from late pregnancy through to the first and second phases of labor. Fuchs 1991 [31] found a maximum pulse frequency of 3 per 10 min in late labor. *Rise of oxytocin levels in connection with birth* A very pronounced rise of oxytocin levels (sometimes as much as a 4-fold increase) was observed in connection with birth of the baby, compared to levels in pregnancy, early labor, or immediately following PLCS: Vasicka 1978 [17], Leake 1981 [19], Goodfellow 1983 [21], Husslein 1983 [22], Kuwabara 1987 [28], Thornton 1988 [29], Oosterbaan 1989 [30]. *Elevation of oxytocin levels in connection with expulsion of the placenta or postpartum* In some studies, elevated oxytocin levels were also found during the third stage of labor, likely in connection with expulsion of the placenta (Kumaresan 1975 [15], Husslein 1983 [22], Thornton 1988 [29], Fuchs 1991 [31])

**Table 5:** Summary of oxytocin levels during pregnancy and in non-pregnant women

In the studies by Kumaresan 1974 [14], Vasicka 1978 [17], Dawood 1979 [18], Otsuki 1983 [20], De Geest 1985 [27], and Kuwabara 1987 [28], a rise of oxytocin levels was recorded with advancing pregnancy. These data demonstrate that oxytocin levels increase during pregnancy. Regardless of the basal levels, and the type of technique used for analysis of oxytocin levels, oxytocin levels increased about 2 to 4-fold. Oxytocin levels in non-pregnant women varied between 1.7 and 11.7 pg/mL.

**Table 6:** Summary of the occurrence of uterine contractions in relation to plasma levels of oxytocin during labour without and with synthetic oxytocin

Dissociation between oxytocin levels and uterine contractions The time relationship between oxytocin levels and uterine contractions was studied in 4 of the articles. In all of these studies, no association was found between oxytocin peaks and uterine contractions: Gibbens 1976 [16], Dawood 1979 [18], Leake 1981 [19], Otsuki 1983 [20]. Effect of synthetic oxytocin administration on oxytocin levels and uterine contractions Infusions of synthetic oxytocin at a rate of 4–6 mU/minute gave rise to a significant rise in oxytocin levels in women at term without labour, which corresponded to the oxytocin levels seen during physiological labour. At an infusion rate of 10–16 mU/min, oxytocin levels were higher than physiological levels (Fuchs 1983 [23]). Doubling the dose lead to doubled levels (Fuchs 1983 [23]), Amico 1984 [24]), Bolus injections of 4–8 mU of synthetic oxytocin in women at term, without labour gave rise to an increase of oxytocin levels of the same size as observed during physiological oxytocin pulses and was associated with uterine contractions. (Fuchs 1991 [31]).	

**Fig. 2:**
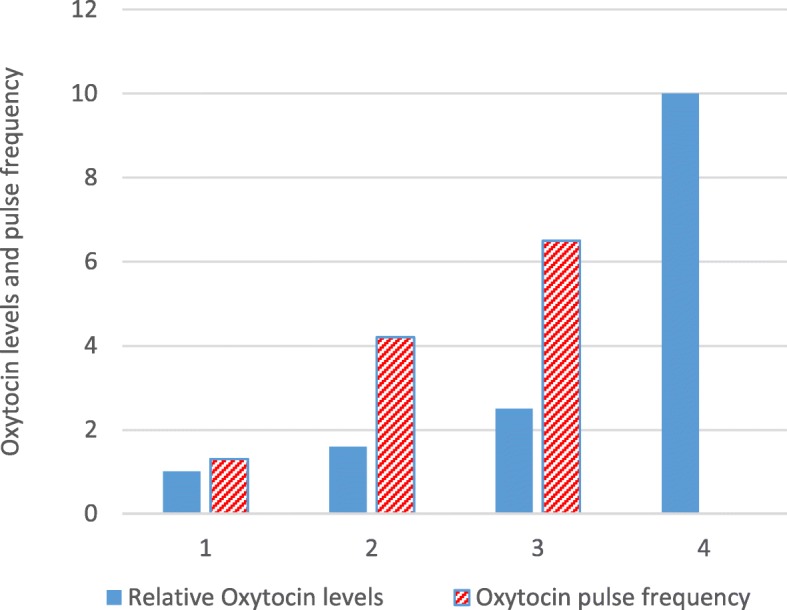
Schematic figure of oxytocin levels (relative values), and oxytocin pulse frequency, averaged over 30 min, in pregnancy and physiological labour and birth. Data compiled from Fuchs 1991 [31] in relation to: term pregnancy (1); first stage labour (2); second stage labour (3). Data in relation to birth (4) compiled from studies cited in Box 1: Rise of oxytocin levels in connection with birth

**Fig. 3:**
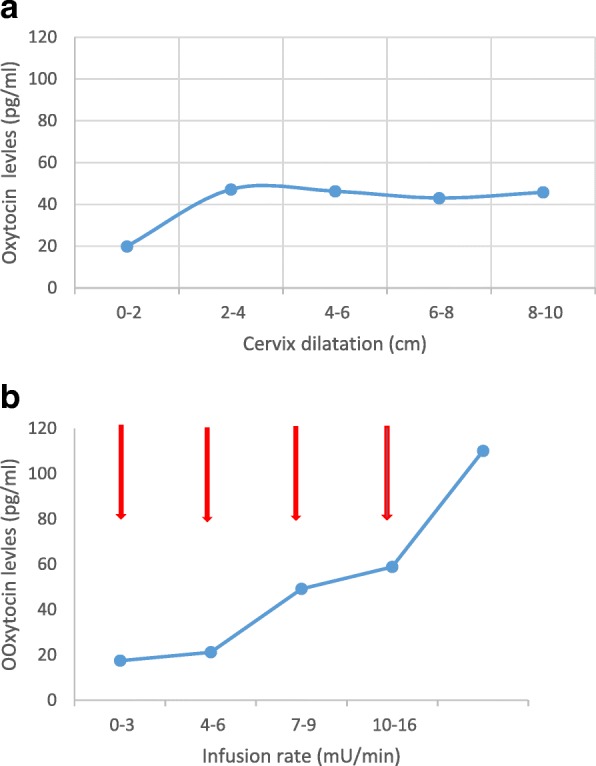
a Oxytocin levels in physiological labour. Means from samples collected 5 times from 17 women over the duration of first stage. Note that peaks are not observed because samples were collected at long intervals. Adapted from Fuchs 1983 [23]. b Oxytocin levels with synthetic oxytocin infusion. Means from samples collected 5 times from 15 women induced with synthetic oxytocin. Samples were collected, as indicated by arrows, before infusion begun at 1-3 mU/min, and at the end of each infusion period (before the next increase). Adapted from Fuchs 1983 [23]

**Fig. 4:**
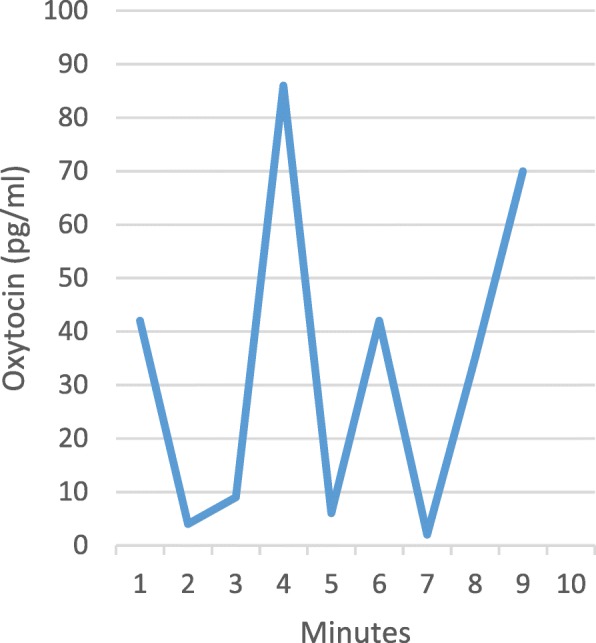
Oxytocin levels in physiological labour. Serial samples collected every 1 min over 10 min from one woman. Adapted from Dawood 1979 [18]

Four schematic figures were created which describe the change of oxytocin levels over time (Figs. 2, 3a, b, and 4). The data used for the figures have been taken from the included studies, as indicated in the text legends of the figures, and have been transformed and simplified for clarity. Figures show oxytocin levels during pregnancy and labour (Fig. 2), the pattern of oxytocin levels during spontaneous labour versus induction of labour with infusion of synthetic oxytocin (Fig. 3a and b), and the very short-lasting character of oxytocin pulses (Fig. 4).

### Extraction of data from the included articles

Data from the included articles has been reported in Tables 1, 2, 3, 4, 5, 6 and Figs. 2, 3a, b, and 4, as described above.

#### Coch et al. 1965 [13]

Serial blood samples were collected from 18 women during physiological birth. “Oxytocin equivalent activity” was demonstrated in samples collected from both the jugular vein (levels ranged between 300 and 900 μU/mL) and a peripheral vein (levels up to 100 μU/mL). The oxytocin equivalent activity increased as labour progressed and reached maximum levels during the second stage of labour, when oxytocin equivalent activity in the jugular vein was up to 8- to 9-fold higher than that in peripheral plasma. After birth, jugular vein oxytocin equivalent activity decreased to levels in peripheral venous blood. The high levels of oxytocin equivalent activity in the jugular vein relative to those in a peripheral vein indicate that oxytocin is released from a central source (the pituitary) during the second phase of labour.

#### Kumaresan et al. 1974 [14]

Multiple blood samples (*n* = 79) were collected at random intervals from 5 women during active physiological labour and birth. Oxytocin levels in plasma averaged 181 μU/mL (range 35–435 μU/mL) in 79 samples. The variation between samples was enormous, indicative of a pulsatile release of oxytocin. The data obtained in this study could not determine whether oxytocin levels were higher during labour than during pregnancy, perhaps due to the wide between-sample variations.

In relation to oxytocin in pregnancy, levels rose from 66 μU/mL to 165 μU/mL between weeks 4 and 40 (*n* = 280). The main rise was observed between weeks 25 and 40. There was a significant correlation between oxytocin levels and weeks of gestation (*p* < 0.01). Oxytocin levels were less than 1.0 μU/mL (*n* = 15) in non-pregnant women. Note that there was a marked difference between oxytocin levels in non-pregnant and pregnant women.

#### Kumaresan et al. 1975 [15]

Serial blood samples were collected from 29 women, from physiological labour and birth to 4 days postpartum. Mean oxytocin levels were approximately the same 10 min before and directly after birth: 90 ± 11 μU/mL and 82 ± 12 μU/mL respectively. Oxytocin levels decreased gradually postpartum (day 1–66 ± 8 μU/mL, day 2–50 ± 9 μU/mL, day 3–54 ± 9 μU/mL and day 4, the same as day 3). The fall in oxytocin levels between day 1 and days 2, 3 and 4 was significant (p < 0.01). Lower oxytocin levels postpartum indicates that oxytocin levels were raised during labour.

#### Gibbens et al. 1976 [16]

Serial blood samples (8) were collected from 97 women during physiological labour and birth. Oxytocin levels were not detected in all samples, likely due to its pulsatile release. A progressive increase in the number of positive results was apparent throughout the first and second stage of labour, suggesting that oxytocin levels increase during the course of labour. Oxytocin levels, indicating the size of pulses, varied between 2 and 12.5 μU/mL. A decrease in oxytocin levels was seen in the third stage of labour. There was no obvious relationship in time between oxytocin levels and the occurrence of uterine contractions.

#### Vasicka et al. 1978 [17]

Serial blood samples were collected from 15 women during physiological labour and birth. No surge of oxytocin was observed at the onset of labour, suggesting that other factors than a rise oxytocin may be responsible for initiation of labour. Oxytocin levels varied between 64 and 148 μU/mL during labour. The most extensive release of oxytocin was seen in connection with maximal cervical and vaginal distention in 12 of the 15 women. The substantial rise of oxytocin levels in connection with birth most likely corresponds to the Ferguson reflex.

Oxytocin levels were also measured in pregnancy, and increased from 126.9 ± 21.1 μU/mL in gestational week 24 to 153.0 ± 26.6 μU/mL in week 40 (*n* = 15). While oxytocin levels rose during pregnancy, the relationship between oxytocin levels and gestational age did not reach statistical significance. Two spikes per patient were observed during gestational weeks 24 to 34 and from 36 weeks progressively increasing oxytocin levels were found in three consecutive samples collected with 1 week intervals.

#### Dawood et al. 1979 [18]

Serial blood samples were collected every minute, during a 10-min period, from 7 women during pregnancy and physiological labour and birth. The release of oxytocin was episodic with a frequency of 2–3 “spurts” (pulses) per 10 min. The levels of oxytocin varied between 0 and 86 pg/mL during labour (*n* = 3). There was no obvious connection in time between oxytocin levels and uterine contractions.

Plasma levels of oxytocin during pregnancy rose from 17.9 ± 2.1 pg/mL (weeks 1–20) to 28.9 ± 4.7 pg/mL (weeks 21–30) and to 32.9 ± 2.9 pg/mL (weeks 31–32) (*n* = 362). There was a highly significant correlation between the mean maternal plasma oxytocin and the week of gestation (*p* < 0.005). A marked increase in maternal oxytocin levels to 74.2 ± 14.2 pg/mL was observed at 39 weeks of gestation, compared to the levels at 38 weeks (26.4 ± 4.8 pg/mL). A significant drop in levels from 39 to 40 weeks of gestation was also found. When sampling was performed every minute, 2–3 pulses per 10 min were observed. The amplitude of the pulses was greater during labour than in samples collected during pregnancy according to the same time schedule.

#### Leake et al. 1981 [19]

Blood samples were collected from 59 pregnant women, and serially from 50 women at the onset, peak and immediately after, individual contractions, at several times during physiological labour and birth. The oxytocin concentrations were 1.3 ± 0.2 μU/mL (*n* = 6) 1.6 ± 0.2 μU/mL (*n* = 14) during the latent and active phases of labour respectively, and 1.1 ± 0.1 μU/mL (*n* = 19) at the time of initial visualization of the fetal head. During birth of the fetal head, there was a significant 4-fold rise in plasma oxytocin to 4.2 ± 1.1 μU/mL (*n* = 11), compared to the levels obtained at the time of initial visualization of the fetal head (*p* < 0.05).

Average oxytocin levels measured during a single uterine contraction during the latent or active phase of physiological birth were 1.5 ± 0;3 μU/mL before, 1.4 ± 0.2 μU/mL at the peak and 2.0 ± 0.3 μU/mL after the contraction. This indicates no immediate correlation in time between plasma oxytocin levels and uterine contractions.

Average oxytocin levels in women between 15 and 42 weeks’ gestation were 1.3 ± 0.1 μU/mL (*n* = 59), and in non-pregnant women, 1.4 ± 0.2 μU/mL (*n* = 102). Plasma oxytocin levels obtained during labour did not differ from the levels obtained during pregnancy or from the levels observed in non-pregnant women, 1.3 ± 0.1 μU/mL, nor was there any significant difference between oxytocin levels in pregnant and non-pregnant women.

#### Otsuki et al. 1983 [20]

Serial blood samples were collected from 2 subjects in the mid trimester, from 4 subjects at term without labour contractions, and from 6 subjects in the first stage of normal, physiological birth. Blood was taken at 10 s intervals over a period of 2–3 min.

The mean level of oxytocin was 12.6 μU/mL during the first stage of physiological labour, with fluctuations and sometimes spurt releases observed. The size of the spurts in active labour varied between 38 and 88 μU/mL. No correlation in time was observed between spurts or fluctuations of oxytocin levels and uterine contractions. The mean level of oxytocin was 12.1 μU/mL during uterine contractions and 11.5 μU/mL during relaxation.

Plasma levels of oxytocin during pregnancy showed a gradual rise from 5 μU/mL at week 20 to 12.5 ± 7.7 μU/mL at term (*n* = 38). There was no difference between the mean level of oxytocin at term (12.5 μU/mL) and during the first stage of physiological labour ((12.6 μU/mL). Almost no fluctuations of oxytocin levels were observed at mid-pregnancy, whereas substantial fluctuations were seen at term. Compared with labour, the oxytocin fluctuations were not small at term, but the pulse phenomenon was more often detected amongst women who were in labour (3/6), compared to those not in labour (1/4).

#### Goodfellow et al. 1983 [21]

Serial blood samples were collected from 10 women at the beginning and at the end of the second stage of physiological birth (crowning of the head). Oxytocin levels at full dilatation were between 7.2 and 46.0 pg/mL and at crowning between 2.3 and 85.0 pg/mL. The rise in oxytocin levels between full dilatation and crowning was significant.

#### Husslein et al. 1983 [22]

Blood samples were collected from 20 women in physiological labour and 14 pregnant women at term but not in labour. Of these, 7 women received an infusion with synthetic oxytocin in a low dosage at the end of the first stage of labour, and directly after birth 10 women received an oxytocin infusion in a high dosage (100-150mIU/min). Plasma oxytocin levels at full cervical dilatation (50.7 ± 10.7 pg/mL) were significantly raised over oxytocin levels at term (18 ± 3.2 pg/mL) and had returned to levels observed at term 30 min postpartum (29.1 ± 9.7 pg/mL). Taken together the data demonstrate a substantial rise of oxytocin levels during birth.

#### Fuchs et al. 1983 [23]

Serial blood samples were collected from 17 women in early physiological labour and 15 women at term in whom induction of labour with synthetic oxytocin was indicated. In both groups the first sample was collected at admission to the labour ward, and four subsequent samples were collected at 1–3 h intervals. An additional blood sample had been collected 1 or 2 weeks before labour in 10 of the 17 women in the physiological labour group. Synthetic oxytocin infusion was begun at a rate of 1–2 mU/min and was increased stepwise every 15 min. Four pregnant women at term but not in labour served as controls. In this group four blood samples were taken2-hour intervals.

During the first stage of physiological labour, the mean concentration of oxytocin was 45.0 ± 3.9 pg/mL. These levels were significantly higher than oxytocin levels obtained in the same women 1–2 weeks before labour (15.4 ± 8.7 pg/mL) and was higher than the mean concentration in the control (pre-labour) group (19.9 ± 3.1 pg/mL). Plasma levels of oxytocin were already raised over control levels in the first sample collected during early labour and remained at approximately the same level throughout the study. In conclusion plasma oxytocin levels recorded during labour were higher than those observed during pregnancy or at term.

In the synthetic oxytocin infusion group, basal levels of oxytocin were 17.4 ± 4.8 pg/mL. Infusions of synthetic oxytocin at a rate of 4–6 mU/min significantly raised oxytocin levels to 49 ± 10.0 pg/mL. Plasma oxytocin levels during physiological labour were comparable to those produced by an infusion of synthetic oxytocin at a rate of 4–9 mU /minute. Infusion rates at 10-16 mU/minute gave rise to oxytocin levels that were twice as high (110 ± 10.9 pg/mL). It was further calculated that endogenous oxytocin is released at a rate of 1 mU/min under basal conditions in pregnant women.

#### Amico et al. 1984 [24]

Serial blood samples were collected from 11 women with “hypocontractile labour” (arrested progress) in the active phase of physiological labour. Synthetic oxytocin was infused in increasing doses from 1 to 5 mU/min and repeated plasma samples were collected before and during the infusions. The level of oxytocin before infusion with synthetic oxytocin was 1.01 ± 0.31 μU/mL.

In relation to women receiving an infusion with synthetic oxytocin, there was a linear relationship between dosage of infusion of synthetic oxytocin and oxytocin levels. A steady state concentration was found by 40 min following infusion rate changes. However, the rate of infusion in individual women’s, oxytocin levels at the time of “adequate contractions” was very variable, ranging from 0.90–5.06 μU/mL. This 5-fold variability suggests significant differences in oxytocin effects, likely reflecting differing numbers and/or binding of oxytocin receptors.

#### Takeda et al. 1985 [25]

Plasma and CSF samples were collected from 11 pregnant women during gestational weeks 7–41 and from 12 women in physiological labour. Plasma levels and CSF levels of oxytocin were 16.1 ± 2.9 μU/mL and 9.7 ± 1.5 μU/mL respectively in pregnant women, and 19.7 ± 3.3 μU/mL and 18.6 ± 2.3 μU/mL in women in labour. Comparing oxytocin levels in women in labour vs pregnancy, levels of oxytocin in the CSF were significantly higher in labour, but plasma levels did not differ from pregnancy to labour. This suggests that there is an increased release of oxytocin into the CSF during labour and that there may be differential control of the release of oxytocin into the circulation and CSF. Plasma levels of oxytocin were 4.8 ± 0.5 μU/mL (*n* = 15) in non-pregnant women.

#### Takagi et al. 1985 [26]

Plasma and CSF samples were collected from 11 women before emergency caesarean section (EmCS), who had uterine contractions, and from 18 women before prelabour caesarean section (PLCS, elective caesarean), who had no uterine contractions.

Mean plasma levels of oxytocin before EmCS and PLCS were 45.2. ±19.6 μU/mL, and 17.1 ± 22.2 μU/mL respectively Mean CSF levels of oxytocin before EmCS and PLCS were 4.9 ± 4.1 μU/mL and 4.1 ± 2.4 μU/mL respectively. Comparing oxytocin levels in women with and without labour contractions, plasma levels were higher in women with uterine contractions, whereas no difference in CSF levels was found in this study. Plasma oxytocin levels in non-pregnant women were 7 ± 5.3 μU/mL (*n* = 7).

#### De Geest et al. 1985 [27]

Repeated blood samples were collected from 10 women during pregnancy and 10 women during physiological labour and birth. No consistent rise of oxytocin levels was observed during labour. Oxytocin levels were 166 ± 29 μU/mL, 179 ± 29μU/mL, 169 ± 25 and 166 ± 30 μU/mL at cervical dilatation < 3 cm, 5–7 cm, and 10 cm and during the second stage of labour respectively.

In pregnancy, oxytocin levels averaged 47, 77, 125 and 136 μU/mL at, 8–12, 20–24, 30–34 and 38–42 weeks of gestation respectively (*n* = 10). The rise of oxytocin levels over time during pregnancy was significant (*p* < 0.01).

#### Kuwabara et al. 1987 [28]

Serial blood samples were collected from 10 pregnant women over the last 14 days before the onset of physiological labour and also 6 h after onset of labour. In addition, maternal blood samples were collected immediately after spontaneous birth (n = 10), PLCS (n = 15) and EmCS (*n* = 5).

Oxytocin levels were around 20 μU/mL at term, with levels the same at 6 h after onset of labour, indicating no rise of oxytocin levels at this time-point, compared to term. Oxytocin levels were 30.6 ± 5.0 μU/ml after physiological labour and birth; 22.6 ± 1.4 μU/mL after PLCS; and 33.6 ± 3.7 μU/mL after EmCS. Maternal oxytocin levels after physiological labour and birth were significantly higher (*p* < 0.05) than following PLCS. These results indicate that oxytocin levels are elevated at the end of labour.

Six pregnant women provided 36 blood samples during weeks 6–41 of gestation. Ten blood samples were collected from normal non-pregnant women.

Oxytocin levels rose progressively during pregnancy, from 4.1 ± 0.5 μU/mL in non-pregnant women (n = 10) to around 16–17 μU/mL at week 40 of gestation, and there was a significant correlation between oxytocin levels and week of gestation (p < 0. 01). There was no change in oxytocin levels during the last 14 days of pregnancy, and there was no diurnal variation.

#### Thornton et al. 1988 [29]

Serial blood samples were collected every 30 s for 15 min during the late second stage of labour, and throughout the third stage of labour in 15 women.

Oxytocin levels were 3.2 (±2) pmol/L in connection with crowning of the fetal head and 6.4 (±2) pmol/L) after birth of the anterior shoulder (p < 0.01). A peak of oxytocin was seen a few minutes later with a mean oxytocin concentration of 11.6 (±1.5) pmol/L. The data show that there is a substantial rise of oxytocin levels in connection with birth. This oxytocin peak occurs after birth rather than before, suggesting that it is a consequence of the Ferguson reflex, activated as the baby is born.

#### Oosterbaan et al. 1989 [30]

Blood samples were collected in 12 women immediately after physiological labour and birth and 11 women after PLCS. Oxytocin levels after physiological labour and birth were 65 ± 9 pg/mL and were significantly higher than those observed after PLCS, 12 ± 9 pg/mL (*p* < 0.009). These results suggest that oxytocin levels are elevated during physiological labour and birth.

#### Fuchs et al. 1991 [31]

Blood samples were collected at 1 min intervals for 30 min in 11 pregnant women at term with a closed cervix who were scheduled for PLCS; in 13 women in the first stage of physiological labour with less than 6 cm cervical dilatation; and in 8 women in the second stage of physiological labour with full dilatation of the cervix.

Plasma levels of oxytocin were below the detection limit, 0.17 μU/mL, in 85% of the samples in the pregnancy group, in 30% of the samples in the first phase of labour and in 16% of the samples collected during the second and third stage of labour. Levels above 0.45 μU/mL were considered pulses. Discrete pulses were observed in all groups and occurred at irregular intervals and had variable amplitudes. All pulses were of short duration, 2–3 min.

The frequency of pulses per 30 min was lowest in the no labour group (1.2 pulses ±0.54); higher in first stage of labour at 4.2 pulses ±0.54; and maximal during the second and third stage of labour at 6.5 pulses ±0.49. The difference in frequency of pulses between the three groups was highly significant.

The mean pulse amplitudes in women not in labour; in first stage; and in second and third stages were 0.91 ± 0.20 μU/mL, 1.10 ± 0.10 μU/mL, and 1.40 ± 0.35/mL respectively. The mean duration of the pulses was also increased in women in labour (1.9 ± 0.21 min) in comparison to women not in labour (1.2 ± 0.14 min).

As a result of the higher amplitude and duration of pulses, the overall mean plasma oxytocin levels were significantly higher in women in labour than in women not in labour. The mean amount of oxytocin released over 30 min (μU/mL) was 0.26 ± 0.041; 0.42 ± 0.04; and 0.65 ± 0.12 in women with no labour; in the first stage of labour; and during the second and third stages of labour (combined) respectively.

Bolus injections of synthetic oxytocin were given to 18 women who were not in labour at 2, 4, 8 and 16 mU dosage. A significant rise of oxytocin was detected in those receiving 4, 8 and 16 mU of synthetic oxytocin. Peaks in oxytocin occurred 30–60 s after injection. The peaks induced by 4 and 8 mU of synthetic oxytocin were of the same size as the physiological peaks during labour (around 1.1microU/mL). These bolus injections stimulated uterine contractions. The average the number of contractions in the first 10 min after the injection was correlated with the mean peak plasma level in each group.

#### Stocche et al. 2001 [32]

Blood samples were collected from women during labour, both before and after administration of two types of analgesia in this randomized, open-label study. Oxytocin levels during the first stage of labour were similar in the two groups of women before administration of analgesia; 7.4 ± 2.1 pg/mL (*n* = 15) and 6.6 pg/mL ±3.1 (n = 15).

## Discussion

### Summary of results

Based on a systematic review on plasma levels oxytocin in physiological labour and birth, we summarize the data as follows: Basal oxytocin levels rise during pregnancy. Oxytocin levels increase during labour. Pulses of oxytocin occur with increasing frequency, amplitude, and duration from the end of pregnancy towards the end of the second stage of labour. The maximal frequency of pulses is 3 per 10 min. A pronounced (four-fold) rise of oxytocin occurs in connection with birth. Oxytocin is also released after birth during the third phase of labour in connection with expulsion of the placenta. Oxytocin pulses are not directly in time associated with uterine contractions. Infusion of synthetic oxytocin at a rate of 4–9 mU/minute gives rise to oxytocin levels equivalent to levels during physiological labour. Oxytocin levels double in response to doubling of the infused dose. Administration of 4–8 mU of synthetic oxytocin as an intravenous bolus injection gives rise to peaks of oxytocin that are equivalent in size to peaks that occur during physiological birth and stimulate uterine contractions. Oxytocin levels in CSF rise during labour and birth.

### Methodological considerations

The studies that are described and analysed in this systematic review are mainly of explorative research design, and may at first sight seem less sophisticated, compared to contemporary randomized controlled studies. However, these studies are of high quality. It is important to realise that measuring oxytocin levels in repeated samples during the long duration of labour and birth is extremely complex, not only because of the intrinsic challenges of sampling blood in a labouring woman, but also because of ethics, expense, and the complex bio-analytic methods that are required.

The design and content of the studies vary considerably, and therefore the studies are difficult to compare in a systematic way, and also a standard quality assessment of the articles was not possible to carry out. The studies included single oxytocin levels obtained during labour, as well as sequences of oxytocin levels obtained during pregnancy and labour, with time intervals varying from weeks to minutes, even seconds. In addition, the included studies used different techniques for oxytocin measurement, giving numerical differences in oxytocin levels, and even different effect patterns. Some of these differences are related to the technology and assays available at the time of the study. Generally, more recent studies have used more accurate measurement tools, as discussed below. Because of these differences, data from articles using different methods cannot be collated.

The first methods used to determine oxytocin levels in blood were based on biological activity. For example, “oxytocin equivalent activity” measured the effect of oxytocin to stimulate milk ejection in lactating rabbits [13]. Such techniques are complicated and relatively unspecific and insensitive. Subsequently, immunological techniques were developed such as radioimmunoassay (RIA), which measures the propensity of the oxytocin in plasma samples to influence the binding between antibodies to oxytocin and radioactive oxytocin. With the development of RIA, increasingly smaller amounts of oxytocin could be measured. However, these techniques are not completely specific, and it is possible that other substances in plasma might influence the binding between oxytocin and its antibodies, and affect the results obtained. Extraction of the plasma samples prior to the assays was introduced to reduce this risk and increase the specificity of the RIA. Another factor that may influence the results is the specificity of the antibodies that react with oxytocin in the radioimmunoassay. This may explain the variation in the included studies, as to basal levels of oxytocin obtained with different assays, but also in the patterns of effect [31].

Enzyme-linked immunosorbent assay (ELISA, EIA) is another type of immunological method used to measure oxytocin levels. Studies using ELISA have obtained very high oxytocin levels, along with different effect patterns, compared to studies using RIA [33]. For example, some studies that used ELISA have found no rise of oxytocin levels during pregnancy [34]. In addition, the validity and reliability of ELISA as a measure for oxytocin levels have been seriously questioned [35].

The timing and frequency of collection of plasma samples is also very important. Even with a specific and sensitive technique for analysis of oxytocin, the short-duration oxytocin pulses in labour require special consideration. Repeated measurements during a limited period of time (sampling at 30 s or 1 min intervals) will allow detection of peaks [31].

The time period during which sampling is performed is also of importance. For example, sampling at several time points during pregnancy is obviously necessary to detect a gestational rise. Likewise, the clear-cut rise of maternal oxytocin levels in connection with birth can only be identified if repeated samples are collected over the duration of labour.

## Discussion of the results

### Oxytocin levels during labour and birth

There is no data suggesting that physiological labour onset occurs as a consequence of a sudden rise of oxytocin levels. Several of the included studies, however, show a gradual rise of oxytocin levels during physiological labour. During the first stage of labour, oxytocin levels are twice as high as before labour onset, as demonstrated in Figs. 2 and 3a. The oxytocin pulses are very short-lasting and may reach quite high concentrations (Fig. 4). As labour progresses, these pulses increase in size and frequency, reaching maximal frequency (3 pulses per 10 min) just before the baby is born (Table 4 and Figs. 2, and 4).

Some of the oxytocin peaks occurring during labour may be spontaneous and of central origin, whereas some of them are likely to be induced in response to activation of the Ferguson reflex. This reflex is stimulated when uterine contractions press the head of the foetus down against the cervix and vaginal wall. Afferent sensory nerve fibres are activated, and send impulses via the spinal cord to the SON and PVN, resulting in the release of oxytocin into both the brain and circulation. This causes a feed-forward effect that accelerates oxytocin release, known as the Ferguson reflex [36]. The maximal expression of this reflex during birth corresponds to the 3- to 4-fold rise of oxytocin levels observed during, and immediately after, birth of the baby (Table 4 and Fig. 2).

### Link between oxytocin and uterine contractions

The uterine muscles are highly sensitive to oxytocin in labour and birth due to upregulation of the sensitivity/concentration of oxytocin receptors by high oestrogen elevations in late pregnancy [37]. In addition, oxytocin release in labour promotes prostaglandin release, further strengthening uterine contractions and labour progress [23, 37]. As little as 4–8 mU given as bolus dose stimulated uterine contractions just before labour onset [31].

It may seem surprising that studies consistently found no temporal connection between uterine contractions and oxytocin peaks, even when sampling was very frequent. This may be explained by the involvement of the autonomic nervous system (ANS) in labour, and especially the parasympathetic nervous system (PSNS) branch. The uterus is innervated by both PSNS and SNS branches of the ANS, with powerful effects on labour and birth. Parasympathetic activation promotes uterine contractility and increases circulation to the uterus and baby. The role of the SNS is more complicated. Sympathetic activation (e.g. from anxiety or fear) may trigger ineffective contractions and inhibit uterine circulation, or may in some cases give rise to long-lasting and more painful contractions [38].

Oxytocin in the brain activates the PSNS. Some oxytocinergic fibres from the PVN reach the parasympathetic networks (plexa) in the lumbosacral region of the spinal cord, where they connect with neurons that stimulate uterine contractions [39]. Thus neurogenic oxytocin may influence and amplify the effects of circulating oxytocin, as the release of oxytocin into the circulation and the activation of the PSNS from the PVN are coordinated.

This two-fold effect of oxytocin on uterine contractions, via circulating oxytocin and via the PSNS, may explain the unexpected finding that the peaks of oxytocin seen during normal labour are not directly in time associated with uterine contractions [16, 19–21].

### Central actions of oxytocin during labour and birth

As discussed in the introduction, oxytocin induces many positive effects by its central actions within the brain. These include stimulation of friendly social interaction, enhanced wellbeing and positive mood, and reduced anxiety, pain and stress [4]. Some of these effects also occur during labour and birth as a consequence of oxytocin released in the brain. Oxytocin is released from dendrites and cell bodies of the magnocellular neurons of the SON and PVN during physiological labour and birth. In addition oxytocin is released from nerves from the parvocellular neurons in the PVN. In this way oxytocin reaches important regulatory areas in the brain, for example areas involved in regulation of social behaviour and fear, pain, stress and wellbeing. Obviously the central effects of oxytocin are not induced by the oxytocin released into circulation but directly from nerves within the brain. In fact oxytocin in the circulation does not pass into the brain because of the blood-brain barrier [4, 40–42].

#### Examples of central oxytocin effects during labour and birth

Oxytocin released within the brain and the spinal cord during labour and birth will decrease the experience of pain. This effect involves release of endogenous opiates. The oxytocin induced pain relief during birth may be linked to the amnesic effect, which helps the new mother forget the intensity of labour [43].

Women can experience strong positive emotions during birth, especially after the baby is born [4, 44, 45]. As shown in several of the included articles, plasma oxytocin levels exhibit a 3–4-fold rise in the circulation as the baby is born. Most likely a parallel rise of oxytocin also occurs in the brain at this point of time, which might be linked to a release of dopamine [4].

Oxytocin released during labour and birth promotes the future interaction between mother and baby and decreases fear and stress levels. After birth, oxytocin helps the mother to bond with her baby, and this effect is reinforced by skin-to-skin contact immediately after birth [4]. In addition, central release of oxytocin in labour enhances sensitivity of the skin, which further facilitates maternal oxytocin elevations during postpartum skin-to-skin contact [46]. At this time, oxytocin also promotes vasodilation of the mother’s chest wall for newborn warming [47].

Given the information above, it is obvious that oxytocin released in the brain during labour and birth exerts many positive and adaptive effects in the mother. Not only is the intensity of pain, fear and stress decreased, but also the mother’s wellbeing is actively stimulated, and future interactions and bonding with her baby are facilitated [4, 41–47].

The central release of oxytocin, and its positive effects, may be modified by environmental factors. Stressful and unfamiliar situations and surroundings may increase stress levels and decrease oxytocin release and PSNS activity. Alternatively, situations perceived as safe, familiar, friendly and supportive by the mother are likely to do the opposite, and promote oxytocin release and PSNS activity. This will facilitate labour progress, as well as the positive central actions caused by oxytocin.

These positive oxytocin effects also require the full functioning of neural pathways involved in physiological labour and birth. These pathways include the Ferguson reflex, which can be effectively be blocked by medical interventions such as epidural analgesia. In this way epidural analgesia may not only reduce oxytocin blood levels and progress of labour, but also central oxytocin levels and adaptive effects [4, 21].

### Levels and effects after infusion of synthetic oxytocin

While the use of synthetic oxytocin was not initially the subject of this review, some of the included studies measured oxytocin levels when synthetic oxytocin was infused. In view of the widespread use of synthetic oxytocin for induction and augmentation of labour, we decided to include this information.

Infusions of synthetic oxytocin for induction or augmentation of labour are generally commenced at a low rate of around 1–3 mU/min, with the dose either increased step-wise (arithmetic) or doubled (geometric) at 15–40 min intervals, although protocols vary widely. Generally the maximum dose of synthetic oxytocin does not exceed 32 mU /min [23, 48].

One of the included studies found that, when synthetic oxytocin was infused in doses up to 9 mU/min, maternal plasma levels of oxytocin were similar to those observed during physiological labour [23] whereas doses around 10–16 mU/min gave rise to levels that were approximately double those of physiological labour (Fig. 3a & b) [23]. This implies that the concentrations obtained following infusion rates over 16 mU/min exceeds physiological levels. A steady state concentration was found by 40 min after a change in infusion rate [24]. It is of interest that administration of a bolus as low as 4–8 mU of synthetic oxytocin gave rise to uterine contractions in women at term, with an elevation of oxytocin levels comparable to levels obtained during a physiological pulse in labour [31].

In contrast to the episodic, narrow peaks of oxytocin in physiological labour, administration of synthetic oxytocin produced flat oxytocin levels in maternal blood. These flat levels may influence the pattern of uterine contractions and contribute to the hyperstimulation that can result from high doses of synthetic oxytocin [48]. Excessive uterine activity may also compromise fetal blood supply, causing hypoxia.

In addition, prolonged exposure to synthetic oxytocin may eventually lead to reduced contractility of the uterine muscles due to desensitization of oxytocin receptors [49]. The decreased efficacy of oxytocin may also increase the risk of postpartum haemorrhage [50]. In addition, the high levels of lactate that accumulate in uterine muscle [51], and hypoxia due to excessive uterine contractions, may also contribute to these risks.

The pharmacokinetics of oxytocin during labour are surprisingly poorly studied. As studied in non-pregnant women, synthetic oxytocin is degraded according to a two-compartment model [52]. However in labour, the levels of oxytocinases, the enzymes that degrade oxytocin, increase 10- to 20-fold, which may also influence the degradation of oxytocin and thereby the half-life and metabolic clearance rate of oxytocin [19, 24, 53].

### Implications for clinical use of synthetic oxytocin in labour and birth

Following on from the findings of our systematic review, an important question is: “How can the administration of synthetic oxytocin in labour and birth imitate physiological patterns and effects, as far as possible?”

From this perspective, a more physiological method of administering synthetic oxytocin would be to use a pulsatile infusion pattern, which is more aligned with labour physiology. Several studies have found that the total dose requirement of oxytocin is 20 to 60% reduced with pulsatile administration compared to continuous infusions. In addition, there is generally less hyperstimulation, and equivalent outcomes for mothers and babies such as caesarean rates and outcomes for the baby [54–61]. In addition, a recent in vitro study has suggested that the pulsatile administration pattern of oxytocin may maintain myometrial receptor sensitivity [62] and therfore maintain uterine contractility.

Another possible conclusion from the recent pharmacokinetic data, which show a relatively longer half-life of oxytocin than currently assumed [52], would be that doses should not be increased with too short intervals. Intervals of 30–40 min rather than 15 min would be consistent with these understandings. Too-short dosing intervals could lead to unnecessarily high cumulative exposures, as levels are increased before the full effects of the previous doses are achieved. In order to achieve the lowest dose exposure, it makes sense to carefully titrate the dose against effect, taking into account this pharmacokinetic data. Arithmetical vs geometric dose increases would be more consistent with low dose exposure.

These hypotheses and models need to be thoroughly studied in relation to the use of synthetic oxytocin in labour, especially given its widespread use.

## Conclusions

This systematic review shows that plasma levels of oxytocin increase gradually during pregnancy and through physiological labour. Short-lasting peaks of oxytocin occur with increasing frequency during labour, and a substantial rise of oxytocin levels occurs when the baby is born. Circulating oxytocin promotes uterine contractions. Infusions of synthetic oxytocin at low doses (less than 10 mU/mín) give rise to similar levels of oxytocin as during physiological labour. Oxytocin released into the brain during labour and birth influences neuroendocrine mechanisms and thereby maternal mood, behaviour, and physiology in a positive way. Infusions of synthetic oxytocin do not give rise to the same beneficial effects in the brain as does endogenous oxytocin released during physiological labour and birth, because oxytocin from the circulation does not pass the blood brain barrier. This implies that labour with synthetic oxytocin infusion does not fully replicate the adaptive effects of physiological labour and birth.

## Additional file

Additional file 1 Search strategy, 6^th^ October 2015. (DOCX 38 kb)

## Data Availability

All information is given in the article and additional files.

## Abbreviations

ANS: Autonomic Nervous System
CNS: Central Nervous System
COST: European Cooperation in Science and Technology
CSF: Cerebrospinal Fluid
EIA: Enzyme Immunoassay
ELISA: Enzyme-Linked Immunosorbent Assay
L: Liter
mg: Milligram
Min: Minute
mL: Milliliter
mM: Millimolar
mU: Milli-unit
ng: Nanogram
nM: Nanomolar
pg: Picogram
pM: Picomolar
PRISMA: Preferred Reporting Items for Systematic Reviews and Meta-Analyses
PSNS: Parasympathetic Nervous System
PVN: Paraventricular Nuclei
RIA: Radioimmunoassay
SNS: Sympathetic Nervous System
SON: Supraoptic Nuclei
μg: Microgram
μM: Micromolar
μU: Micro-unit
