# Supplementary material for: Maternal plasma levels of oxytocin during physiological childbirth – a systematic review with implications for uterine contractions and central actions of oxytocin
Source: BMC Pregnancy Childbirth. 2019 Aug 9;19:285. doi: 10.1186/s12884-019-2365-9 (PMC6688382; doi:10.1186/s12884-019-2365-9)
Supplement: Supplementary file 1 — Search strategy, 6th October 2015. (DOCX 38 kb) [file 12884_2019_2365_MOESM1_ESM.docx]

**Additional file 1**

**Search strategy, 6^th^ October 2015**

**PubMed:**

(((Women[Title/Abstract] OR maternal[Title/Abstract] OR mother[Title/Abstract] OR parturient[Title/Abstract])) AND (Oxytocin[Title/Abstract] OR oxytocin release[Title/Abstract] OR oxytocinase release[Title/Abstract] OR oxytocin receptor*[Title/Abstract] OR endogenous oxytocin[Title/Abstract] OR oxytocin blood level*[Title/Abstract] OR plasma level*[Title/Abstract] OR amniotic fluid[Title/Abstract] OR amniotic liquor)) AND (labo*r[Title/Abstract] OR *birth[Title/Abstract] OR parturition[Title/Abstract] OR postpartal[Title/Abstract] OR postpartum[Title/Abstract]) Filters: Humans

**CINAHL:**

(Women OR maternal OR mother OR parturient) AND (Oxytocin OR oxytocin release OR oxytocinase release OR oxytocin receptor* OR endogenous oxytocin OR oxytocin blood level* OR plasma level* OR amniotic fluid OR amniotic liquor) AND (labo*r OR *birth OR parturition OR postpartal OR postpartum)

**PsycINFO:**

(Women OR maternal OR mother OR parturient) AND (Oxytocin OR oxytocin release OR oxytocinase release OR oxytocin receptor* OR endogenous oxytocin OR oxytocin blood level* OR plasma level* OR amniotic fluid OR amniotic liquor) AND (labor OR birth OR parturition OR postpartal OR postpartum)
